# HuangQin Decoction Attenuates CPT-11-Induced Gastrointestinal Toxicity by Regulating Bile Acids Metabolism Homeostasis

**DOI:** 10.3389/fphar.2017.00156

**Published:** 2017-03-30

**Authors:** Xu Wang, Dong-ni Cui, Xiao-min Dai, Jing Wang, Wei Zhang, Zun-jian Zhang, Feng-guo Xu

**Affiliations:** ^1^Key Laboratory of Drug Quality Control and Pharmacovigilance, Ministry of Education (MOE), China Pharmaceutical UniversityNanjing, China; ^2^State Key Laboratory of Natural Medicine, China Pharmaceutical UniversityNanjing, China; ^3^State Key Laboratory for Quality Research in Chinese Medicines, Macau University of Science and TechnologyMacau, China

**Keywords:** irinotecan (CPT-11), HuangQin Decoction, bile acids, LC-MS/MS, metabolomics

## Abstract

Irinotecan (CPT-11) is a potent chemotherapeutic agent, however, its clinical usage is often limited by the induction of severe gastrointestinal (GI) toxicity, especially late-onset diarrhea. HuangQin Decoction (HQD), commonly used for the treatment of GI ailments, has been proved could significantly ameliorate the intestinal toxicity of CPT-11. To reveal the mechanisms of CPT-11-induced toxicity and the modulation effects of HQD, a previous untargeted metabolomics study was performed and the results indicated that HQD may protect the GI tract by altering the metabolism of bile acids (BAs). Nevertheless, the untargeted assays are often less sensitive and/or efficient. In order to further confirm our previous findings, here in this paper, serum and tissues metabolic profiles of 17 BAs were analyzed using liquid chromatography-tandem mass spectrometry based targeted metabolomics. The results indicated that serum and tissues levels of most BAs were significantly decreased after CPT-11 administration, except some hydrophobic BAs. Co-treatment with HQD could markedly attenuate CPT-11-induced GI toxicity and reverse the alterations of hydrophobic BAs. Despite the fact that the BAs pool size remained unchanged, the balance of BAs had shifted leading to decreased toxicity after HQD treatment. The present study demonstrated for the first time that the precise interaction between HQD, CPT-11-induced intestinal toxicity and BAs’ homeostasis.

## Introduction

Irinotecan (CPT-11), a water-soluble derivative of camptothecin, is a highly effective chemotherapeutic agent which has been widely used in clinical practice to treat colon (Mukai et al., 2009), gastric (Takiuchi, 2009), pancreatic (Stathopoulos et al., 2003), and varieties of other cancers (Mrozek et al., 2008; Otsuka et al., 2015; Trafalis et al., 2016) through the inhibition of DNA topoisomerase I. However, dose-limiting toxicities myelosuppression, neutropenia, and gastrointestinal (GI) toxicity, particularly severe late-onset diarrhea, compromise the efficacy and safety of irinotecan-based chemotherapy (Kakolyris et al., 2001; Benson et al., 2004). Prophylaxis with atropine inhibit the activity of cholinesterase to eliminate acute (early-onset) diarrhea (Yumuk et al., 2004). However, high dose of loperamide, recommended to relieve CPT-11-induced severe late-onset diarrhea, is non-specific and shows limited effect (Suzuki et al., 2000; Barbounis et al., 2001; Pro et al., 2001). Find out other more effective modulator agents that either alleviate the toxic side effects associated with CPT-11 treatment and/or enhance the therapeutic efficacy of the anti-tumor activity will be of great value.

HuangQin Decoction (HQD), a traditional Chinese formulation from *Shang Han Lun* of the Han Dynasty, has been established for more than 1800 years for the treatment of abdominal spasms, diarrhea, vomiting, and nausea, the same symptoms commonly observed toxic effects in cancer patients receiving chemotherapy (Sharma et al., 2005). Some animal experiments and clinical trials have demonstrated that PHY906 (US Patent No. 7025993), a modified pharmaceutical preparation derived from HQD, could significantly ameliorate chemotherapy-induced GI toxicity and intensify the therapeutic efficacy of a number of anticancer drugs (Yen et al., 2009; Lam et al., 2010, 2015; Saif et al., 2010, 2014; Kummar et al., 2011). However, the underlying mechanisms that are responsible for the modulation effects and molecular process of HQD are still incomprehensive.

HQD is a four-herb Chinese Medicine Formula, including *Scutellaria baicalensis* Georgi, *Glycyrrhiza uralensis* Fisch, *Paeonia lactiflora* Pall, and *Ziziphus jujuba* Mill. The chemical components of HQD usually act simultaneously and synergistically on multiple targets in the body based on holistic concepts. Metabolomics, a novel holistic approach to study metabolic changes, is consistent with the spirit of Traditional Chinese Medicine (TCM). Focusing on endogenous metabolites will provide a global insight into the actions of HQD. Previously, an untargeted metabolomics study using gas chromatography-mass spectrometry (GC/MS) and ultra-fast liquid chromatography coupled with ion trap time-of-flight mass spectrometry (UFLC-IT–TOF/MS) in combination was performed. The study demonstrated that HQD is really a kind of effective modulators of CPT-11. Meanwhile, the potential endogenous metabolites changes associated with CPT-11-induced perturbations and HQD treatment were screened out (Wang et al., 2015). In addition to the perturbations of amino acids, lipids, and energy metabolism homeostasis, the significantly altered bile acids (BAs) could also be considered as potential biomarkers for the metabolic regulatory effects of CPT-11 or HQD. Noting that changes of BAs profiles are extensively linked to CPT-11 exposure (Kobayashi et al., 2001; Sawano et al., 2015; Fang et al., 2016). Moreover, BAs have been proved as sensitive biomarkers to evaluate the intestinal function (Gnewuch et al., 2009; Wildenberg and van den Brink, 2011; Devkota et al., 2012; Zhou et al., 2014). Elucidating the physiological function of BAs could help us understand the mechanisms associated with toxicity and disease state. However, the precise information on the interaction between HQD, CPT-11-induced intestinal toxicity and BAs’ homeostasis is lacking.

Although the altered BAs profiles in CPT-11-induced diarrhea model have been found based on our previous metabolomics analysis, the untargeted assays are often less sensitive and/or efficient. In addition, the metabolites identified by database or library search are ambiguous. Whether one of the mechanisms of HQD’s ability in attenuating CPT-11-induced intestinal toxicity is by restoring the homeostasis of BAs still unclear. Thus in order to further confirm our previous findings, here in this paper, serum and tissues metabolic profiles of 17 BAs were analyzed using liquid chromatography-tandem mass spectrometry (LC-MS/MS)-based targeted metabolomics.

## Materials and Methods

### Chemicals and Reagents

Cholic acid (CA), glycocholic acid (GCA), taurocholic acid (TCA), chenodeoxycholic acid (CDCA), glycochenodeoxycholic acid (GCDCA), taurochenodeoxycholic acid (TCDCA), ursodeoxycholic acid (UDCA), glycoursodeoxycholic acid (GUDCA), tauroursodeoxycholic acid (TUDCA), deoxycholic acid (DCA), glycodeoxycholic acid (GDCA), taurodeoxycholic acid (TDCA), taurolithocholic acid (TLCA), and cortisone acetate were purchased from Sigma-Aldrich (St. Louis, MO, USA). α-Muricholic acid (α-MCA) and tauromuricholic acid (TMCA) were purchased from Steraloids Inc (Newport, RI, USA). All other reagents and solvents were of high performance liquid chromatography (HPLC) grade. Deionized water was purified using a Milli-Q system (Millipore, Bedford, MA, USA). *S. baicalensis* Georgi (Hebei Province), *G. uralensis* Fisch (Inner Mongolia of China), *P. lactiflora* Pall (Anhui Province), and *Z. jujuba* Mill (Henan Province) were authenticated by Dr. Ehu Liu (State Key Laboratory of Natural Medicine, China Pharmaceutical University, China).

### CPT-11 and HQD Preparation

CPT-11 (Jaripharm Inc, Lianyungang, China) was obtained as irinotecan hydrochloride. It was prepared, according to manufacturer’s (Camptosar^TM^, Pfizer Inc., USA) directions and the published papers, to duplicate the clinical formulation: 20 mg/mL CPT-11, 45 mg/mL sorbitol (Sigma, St Louis, MO, USA), and 0.9 mg/mL lactic acid (Sigma, St Louis, MO, USA) dissolved in deionized water and subsequently heated to 70–90°C until the solution turned to clear. The pH was adjusted to 3.5 using 1 M NaOH. The resulting solution was sterile-filtered and stored protected from light until the moment of administration (Hardman et al., 1999; Trifan et al., 2002; Yang et al., 2006).

HQD was prepared by decocting *S. baicalensis* Georgi, *G. uralensis* Fisch, *P. lactiflora* Pall, and *Z. jujuba* Mill at ratio of 3:2:2:2 by dry weight. The filtrate was collected then concentrated as a decoction of 1 g/mL crude drug, stored in the refrigerator before intragastric administration to rats.

### Animal Experiments and Sample Collection

Male Sprague-Dawley rats (Sino-British SIPPR/BK Lab Animal Ltd., Shanghai, China) initially weighing 200 ± 20 g were used in present study. Animals were housed in a routine laboratory conditions with a temperature-controlled (25°C) environment and a 12 h dark/light cycle throughout the acclimatization and experimental periods. The experimental protocols were in accordance to the Guide for the Care and Use of Laboratory Animals and approved by the Animal Ethics Committee of China Pharmaceutical University (License Number: SYXK 2012-0035). After 1 week adaptation, rats were randomly divided into three groups either received vehicle alone, CPT-11 alone, or CPT-11 and HQD. The toxicity model was constructed by treatment of animals with CPT-11 at 150 mg/kg by intravenous injection (i.v.) once a day for two consecutive days (Trifan et al., 2002). Animals in control group injected with the same dose of vehicle. HQD was given orally twice a day from 1 day before the start of CPT-11 injection (day 1) for six consecutive days at a dose of 10 g/kg. While animals in control and CPT-11 group received equivalent Milli-Q water. All the gavage were 30 min prior to the dosing of CPT-11 on days 1 and 2. The mortality, weight loss, and food intake of animals were monitored daily. Scoring of diarrhea was conducted twice daily (8 a.m. and 8 p.m.). The severity of delayed diarrhea was scored as described by Kurita et al. (2000).

**FIGURE 1:**
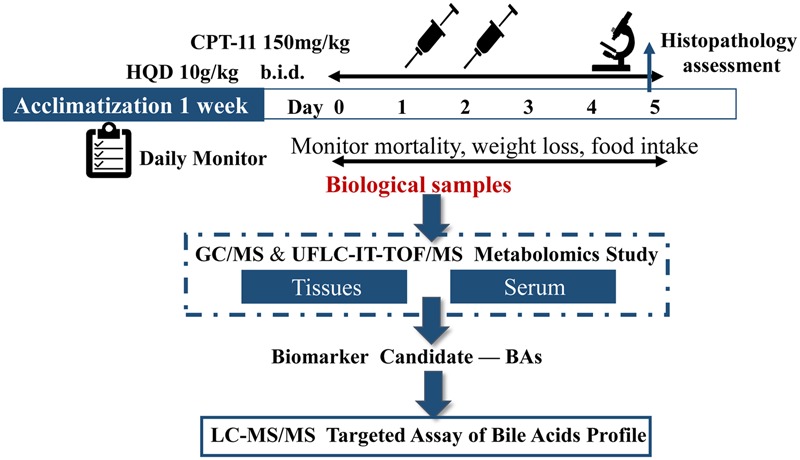
**Experimental design.** Animals either received vehicle alone, CPT-11 alone, or CPT-11 and HQD. The toxicity model was constructed by treatment of animals with CPT-11 at 150 mg/kg by intravenous injection (i.v.) once a day for two consecutive days (day 1–2). HQD (10 g/kg) was given orally twice a day for 6 days beginning on day 0 (day 0–5). The mortality, weight loss and food intake of animals were monitored daily. The rats were finally euthanized on day 5. Serum and tissues (liver, jejunum, ileum, cecum, colon, and rectum) were collected for preparation and analysis. The dashed part is completed in the pilot study.

Diarrhea observed immediately (occur within 2 h) after the dosing of CPT-11 and approximately 48 h after the final administration were considered to be acute diarrhea and late-onset diarrhea, respectively. Serum and tissues (liver, jejunum, ileum, cecum, colon, and rectum) were collected on day 5, the most severe day of late-onset diarrhea. After coagulation (1 h) and centrifugation (8,000 rpm for 10 min), serum samples were aliquoted and then stored at -80°C until needed for preparation and analysis. Some tissue samples were used for histological examination and others were used for quantitative analysis. The scheme of the whole experiment is shown in **Figure 1**.

### Histopathology Examination

Tissue sections (liver, jejunum, ileum, cecum, and colon) were dissected on the most severe day of late-onset diarrhea (day 5), fixed in 10% neutral formalin and stained with hematoxylin–eosin. The routine histopathology assessment was performed in a blinded manner.

### Quantification of Bile Acids in Biological Samples

Metabolomic profiling of different classes of BAs in biological samples was assayed by a LC-MS/MS-validated method. Serum samples were thawed at room temperature. A total of 200 μL methanol and 10 μL cortisone acetate [internal standard (IS), 100 μg/mL] were added to 50 μL serum and the mixture was vortexed for 5 min. After centrifuged twice at 16,000 rpm (4°C) for 10 min, the supernatant was used for LC-MS/MS analysis. Approximately 100–150 mg tissue samples, including liver and intestines, were homogenized in 10 volumes of methanol. After 10 min of centrifugation (16,000 rpm, 4°C), 50 μL supernatant was spiked with 10 μL IS and 150 μL methanol, vortexed, centrifuged, and then used for further analysis. The separation was finally carried out on a ZORBAX Eclipse XDB-C18 (2.1 × 150 mm, 3.5 μm) (Agilent Technologies). The mobile phase consisted of (A) acetonitrile and (B) 0.1% formic acid. The gradient elution started with 25% A for 20 min, linearly increased to 60% B in 65 min and brought back to 25% A in 5 min followed by 10 min of re-equilibration. The mass spectrometry (MS) analysis was performed in a triple quadruple TSQ Quantum mass spectrometer via electrospray ionization (ESI) interface (Thermo Fisher, Palo Alto, CA, USA). The parameters were as follows: ESI (-), spray voltage, 3.8 kV; capillary temperature, 380°C; scan width for multiple-reaction monitoring (MRM), 0.1 *m/z*. Nitrogen was used as sheath (40 arb) and auxiliary (25 arb) gas. Argon was used as the collision gas (1.0 mTorr). BAs with no reference (β-MCA and ω-MCA) were semi-quantified by using calibration curve of homologous standards (α-MCA). The main parameters for MS/MS detection of BAs as well as the internal standard cortisone acetate are summarized in **Table 1**.

**Table 1:** Structures of unconjugated, glycine- and taurine-conjugated bile acids and their optimum MS–MS parameters.

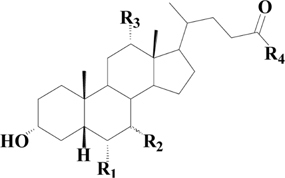							
**Bile acid**	**R1**	**R2**	**R3**	**R4**	**[M-H]^-^**	**MRM transitions**	**CE(V)**
α-MCA	OH (β)	OH(α)	H	OH	407.2	407.2 → 407.2	20
β-MCA	OH (β)	OH (β)	H	OH	407.2	407.2 → 407.2	20
ω-MCA	OH (α)	OH (β)	H	OH	407.2	407.2 → 407.2	20
CA	H	OH (α)	OH	OH	407.2	407.2 → 407.2	20
CDCA	H	OH (α)	H	OH	391.2	391.2 → 391.2	20
DCA	H	H	OH	OH	391.2	391.2 → 391.2	20
UDCA	H	OH (β)	H	OH	391.2	391.2 → 391.2	20
GCA	H	OH (α)	OH	NHCH_2_CO_2_H	464.2	464.2 → 74.0	40
GCDCA	H	OH (α)	H	NHCH_2_CO_2_H	448.2	448.2 → 74.0	40
GDCA	H	H	OH	NHCH_2_CO_2_H	448.2	448.2 → 74.0	40
GUDCA	H	OH (β)	H	NHCH_2_CO_2_H	448.2	448.2 → 74.0	40
TMCA	OH (α)	OH (α)	H	NHCH_2_CH_2_SO_3_H	514.2	514.2 → 80.0	70
TCA	H	OH (α)	OH	NHCH_2_CH_2_SO_3_H	514.2	514.2 → 80.0	70
TCDCA	H	OH (α)	H	NHCH_2_CH_2_SO_3_H	498.2	498.2 → 80.0	70
TDCA	H	H	OH	NHCH_2_CH_2_SO_3_H	498.2	498.2 → 80.0	70
TUDCA	H	OH (β)	H	NHCH_2_CH_2_SO_3_H	498.2	498.2 → 80.0	70
TLCA	H	H	H	NHCH_2_CH_2_SO_3_H	482.2	482.2 → 80.0	70
IS					401.2	401.2 → 340.5	40

*Backbone and side chain structures of the major BAs. CE: collision energy; IS (internal standard): cortisone acetate.*

### Method Validation

The method used in this study was developed in terms of linearity, accuracy, precision, extraction recovery, matrix effects, and stability following the compliance criteria described by Food and Drug Administration (FDA) guidance for Bioanalytical Method Validation. Charcoal-treated serum and tissues homogenates were served as biological matrices for the preparation of standards and quality control (QC) samples. Calibration curves were constructed by plotting the peak–area ratio of each BA to IS versus the concentrations of the BA in each corresponding matrix. Intraday and interday accuracy and precision were determined using 3QC concentrations distributed throughout the calibration range for each analyte. Extraction recoveries only took into account the efficiency of the extraction procedure. Matrix effects were determined by adding the analytes to each blank matrix after extraction and compared with the same amount of analytes in pure solvents. The freeze–thaw, short-term, long-term, and post-preparative stability of the BAs were also investigated in current method.

### Data Processing and Statistical Analysis

All the results are present as mean ± standard deviation (SD). The multivariate data analysis, principal component analysis (PCA) and partial least squares discriminant analysis (PLS-DA), were conducted by SIMCA-P (version 13.0, Umetrics, Sweden). Statistical evaluations were performed using non-parametric Mann–Whitney *U*-test (SPSS Inc., Chicago, USA). Differences were considered the results with *P*-values <0.05.

## Results

### Method Validation

A fully validated method is appropriate for all the determinations. The accuracy ranges from 86.85 to 111.81% in all tested samples. Precision, measured as coefficient of variation (CV), was below 14.37 and 13.30% for intraday and interday for all BAs, respectively. In terms of linearity, the regression coefficients for all the calibration curves of BAs were higher than 0.99. Extraction recoveries at all QC concentrations were >83.42% in serum, >71.79% in liver, and >78.46% in intestine. No significant matrix effects for BAs were observed. In addition, stability studies indicated that BAs were stable in serum and tissues for at least 8 h at room temperature, at least three freeze–thaw cycles, in the -80°C for 2 weeks, and also in the reconstitution solution at 16°C for 12 h.

### Histology Analysis

**FIGURE 2:**
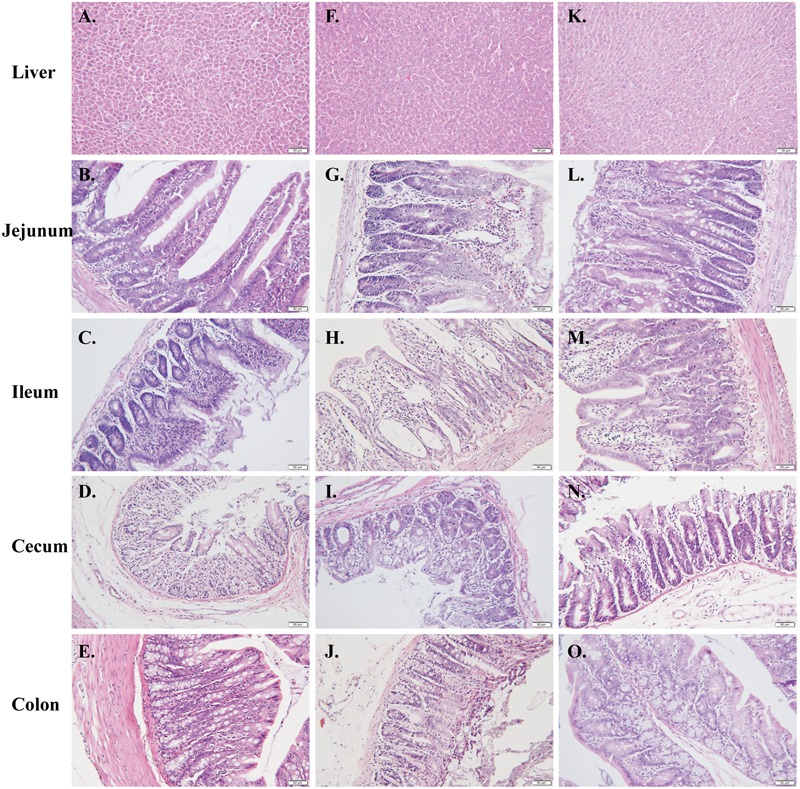
**CPT-11-induced intestinal damage.** Tissue sections (liver, jejunum, ileum, cecum, and colon) were dissected for routine histopathology assessment. Representative photographs of histological examination for **(A–E)** control, **(F–J)** model, and **(K–O)** HQD group. Bar = 50 μm.

As anticipated, marked intestinal pathological changes were observed in rats received CPT-11 alone, including epithelial degeneration, villus shortening, gland atrophy, inflammatory cellular infiltration, and submucosal edema. The histologic lesions were extremely severe in ileum, followed by cecum, and minimal in jejunum and colon. Conversely, liver histology appeared normal. These intestinal damages were significantly alleviated in rats co-treated with HQD (**Figure 2**). Histological sections verified that the CPT-11-induced toxicity model was successfully produced in present study and co-treatment with HQD effectively ameliorated the intestinal damages induced by CPT-11.

### HQD Attenuated the Body Weight Loss and Late-Onset Diarrhea Induced by CPT-11

Rats repeated dosing CPT-11 (150 mg/kg/day i.v.) for two consecutive days appeared serious anorexia and rapid decrease in body weight, reached 85.57 ± 2.20% of initial body weight by day 5. However, rats co-administered with HQD significantly attenuated body weight loss (91.49 ± 2.54% by day 5) and had a higher food intake level as compared with rats only received CPT-11. In addition, severe acute (occurred within 2 h after CPT-11 injection on days 1–2) and late-onset (chronically present during days 4–5) diarrhea were observed in rats treated with CPT-11 alone. Rats co-administered with HQD significantly improved the late-onset diarrhea induced by CPT-11, but failed to prevent the acute diarrhea on days 1 and 2. As expected, rats received the vehicle alone did not show any significant signs of toxicity. None of the rats died during the experiment (**Table 2**).

**Table 2:** Relative body weight and average diarrhea score of animals.

Treatment group	Diarrhea score, mean% ± SD	Relative body weight, mean% ± SD
Control	0.00 ± 0.00	103.35 ± 2.10
CPT-11	2.19 ± 0.99^∗∗^	85.57 ± 2.20^∗∗∗^
CPT-11 + HQD	1.38 ± 0.69^#^	91.49 ± 2.54^###^

*Relative body weight was calculated relative to animals’ weight at the beginning of the experiment (day 0). Significance compared with control group: ^∗∗∗^*P* < 0.001, ^∗∗^*P* < 0.01. Significance compared with model group: ^###^*P* < 0.001, ^#^*P* < 0.05 (Mann–Whitney *U*-test).*

### Targeted Quantitation Revealed Altered Bile Acids Profiles in Multiple Compartments of Rats after CPT-11 Treatment

**FIGURE 3:**
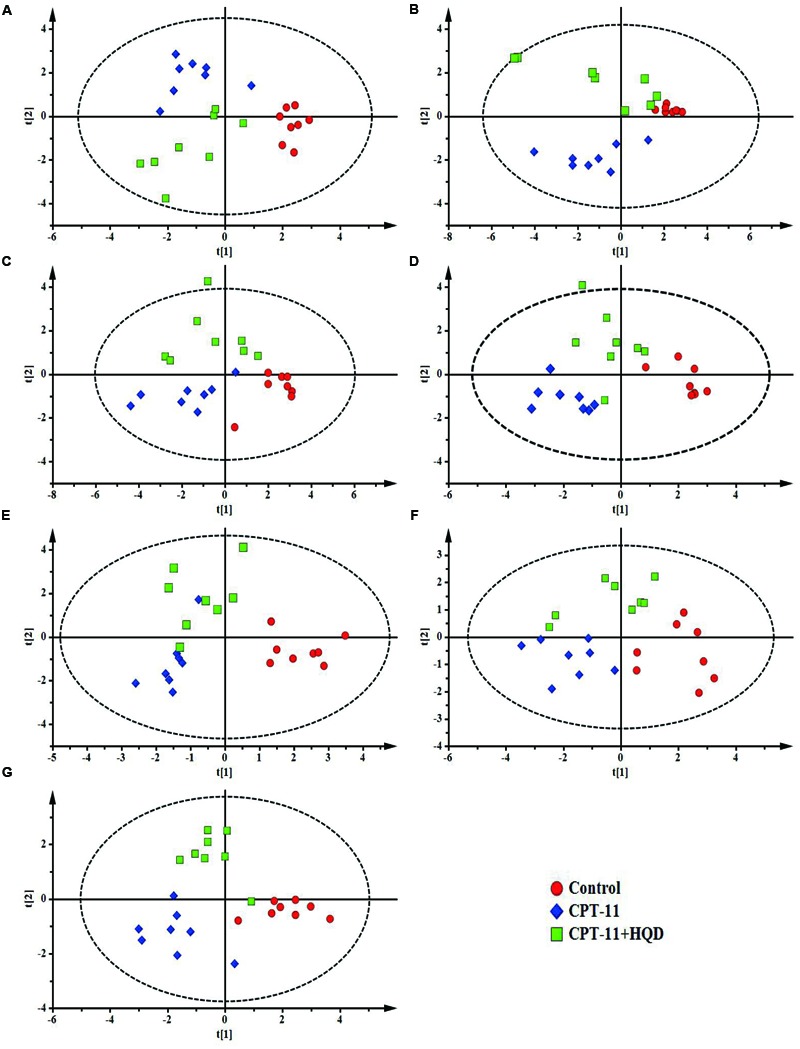
**PLS-DA models of bile acids profiles.** Based on the score plots, bile acids profiles of three groups of rats were shown for **(A)** serum, **(B)** liver, **(C)** jejunum, **(D)** ileum, **(E)** cecum, **(F)** colon, and **(G)** rectum. **(A)** *R*^2^*X* = 0.614, *R*^2^*Y* = 0.806, *Q*^2^ = 0.641; **(B)** *R*^2^*X* = 0.710, *R*^2^*Y* = 0.753, *Q*^2^ = 0.575; **(C)** *R*^2^*X* = 0.530, *R*^2^*Y* = 0.647, *Q*^2^ = 0.483; **(D)** *R*^2^*X* = 0.513, *R*^2^*Y* = 0.830, *Q*^2^ = 0.524; **(E)** *R*^2^*X* = 0.471, *R*^2^*Y* = 0.709, *Q*^2^ = 0.547; **(F)** *R*^2^*X* = 0.614, *R*^2^*Y* = 0.753, *Q*^2^ = 0.540; **(G)** *R*^2^*X* = 0.711, *R*^2^*Y* = 0.875, *Q*^2^ = 0.612.

To targeted assay of BAs profiles throughout the enterohepatic system, concentrations of 17 individual BAs, including seven free BAs, six taurine-conjugated BAs, and four glycine-conjugated BAs were simultaneously determined and quantified in biological samples using a sensitive high-throughput LC-MS/MS (Supplementary Figure 1). A previously uncharacterized compartment-specific dysregulation feature of BAs was identified in different groups of rats. CPT-11-treated animals showed very different profiles from controls, with a marked reduction of CA, TCA, α-MCA, TMCA, UDCA, and TUDCA in serum. Levels of TCA and TUDCA were also decreased in liver and certain intestinal segments. Additionally, β-MCA was increased in liver and colon, while its conjugate—TMCA, was decreased in ileum, colon, and rectum. Notably, the levels of hydrophobic BAs—CDCA, GCDCA, TCDCA, especially DCA, GDCA, TDCA, and TLCA were significantly increased in almost all of the tested tissues. Contrary to what we expected, the most obvious disturbance of BAs homeostasis was shown in serum rather than in target organs, such as liver and intestine. This may be because circulating BAs comprehensively reflected the hepatic input and intestinal reabsorption (Supplementary Table 1). Meanwhile, as shown in PCA and PLS-DA score plots, the CPT-11-treated group serum and tissues could be clearly distinguishable from the control group (**Figure 3** and Supplementary Figure 2).

### HQD Shifted the Balance of Bile Acids Leading to Decreased Toxicity

**FIGURE 4:**
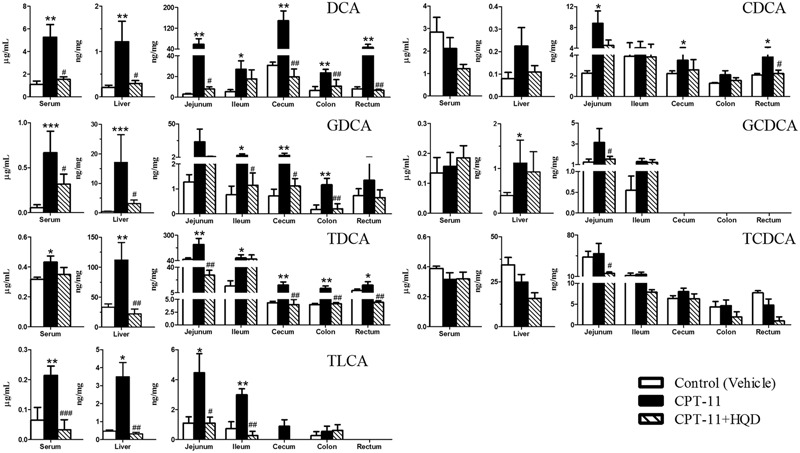
**Concentrations of hydrophobic bile acids in biological samples among different groups.** Graphics represent the mean ± SD. *P*-values were determined by Mann–Whitney *U*-test. Significance compared with control group: ^∗∗∗^*P* < 0.001, ^∗∗^*P* < 0.01, ^∗^*P* < 0.05. Significance compared with model group (only CPT-11-treated group): ^###^*P* < 0.001, ^##^*P* < 0.01, ^#^*P* < 0.05.

Compared with only CPT-11-treated animals, HQD co-treatment could partly restore the homeostasis of BAs. Although there were some overlaps among three groups in PCA score plots (Supplementary Figure 2), the control group, CPT-11-treated group, and HQD co-treated group were separately clustered and could be clearly distinguished in rat serum and tissues based on PLS-DA model (**Figure 3**). The quantitative results showed that HQD mainly regulated the hydrophobic BAs (**Figure 4**). Hydrophobicity is an important determinant of the biological activity exhibited by BAs (Powell et al., 2001). Although the levels of cytoprotective UDCA and its conjugates remained unchanged, that of cytotoxic BAs, including CDCA, GCDCA, TCDCA, DCA, GDCA, TDCA, and TLCA, were markedly decreased in multiple compartments of rats received HQD. In fact, both CPT-11-treated and HQD co-treated rats had a slightly altered BAs pool sizes in serum and tissues. However, there was no statistically significant difference in BAs pool size between CPT-11 group and HQD group (Supplementary Figure 3). These data may reflect the fact that HQD was more likely to shift the balance of BAs rather than influence the BAs pool size. Meanwhile, it also suggested that, in addition to total BAs concentration, the composition of BAs is more important regarding BAs-related functions in the body. The profound remodeling of the BAs composition may be one of the mechanisms of HQD to reduce the toxicity of CPT-11.

## Discussion

**FIGURE 5:**
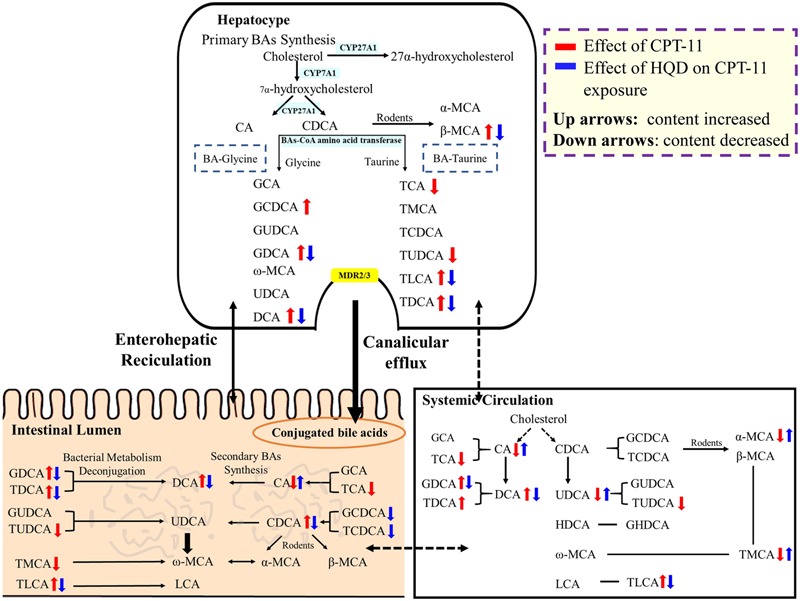
**The impact of CPT-11 and HQD on bile acids metabolome.** The bile acids profile in the intestine shows the combined data from five segments (jejunum, ileum, cecum, colon, and rectum). Bile acids with arrow is significantly changed (↑ content increased; ↓ content decreased).

BAs, the end products of cholesterol metabolism, are major components of bile. They are synthesized exclusively in the liver and further delivered to the intestinal tract, where they are metabolized by the gut microbiota into the secondary BAs. In addition to digesting and absorbing of lipids, BAs are also important signaling molecules that are involved in regulation of various metabolic processes via activation of some specific nuclear receptors and G protein-coupled receptor (Nguyen and Bouscarel, 2008; Prakash and Gorfe, 2013; de Aguiar Vallim et al., 2013; Legendre et al., 2014; Qi et al., 2015). Researches suggested BAs are closely related to intestinal dysbiosis. Thus, elucidating the function of BAs could facilitate our understanding of the mechanisms associated with disease state. To our knowledge, this is the first study that focused on the precise interaction between HQD, CPT-11-induced intestinal toxicity, and BAs’ homeostasis (**Figure 5**).

Multiple factors contribute to the abnormal composition or delivery of BAs. For example, gastrointestine injury hampers the efficient absorption of BAs and thus influences the enterohepatic circulation of BAs. Disturbed intestinal microbiota results in quantitative and qualitative BAs changes. In fact, CPT-11 not only induce intestinal damage, but also disrupt intestinal microbiota (Stringer et al., 2008). Targeted quantitation revealed that CPT-11-treated rats had very different profiles from controls, accompanying with a significantly elevation of CDCA, DCA, and their glycine or taurine conjugates. However, high levels of BAs in lumen, especially those hydrophobic BAs, probably induce or aggravate intestinal damage. Numerous studies have demonstrated that hydrophobic BAs can induce membrane perturbation, disrupt membrane integrity, and release intracellular constituents (Rodrigues et al., 1999; Im and Martinez, 2004; Akare and Martinez, 2005). Fukumoto et al. (1999) have reported that elevation of TDCA, TCDCA, and their unconjugated BAs in serum could enhance gastric mucosal damage and this effect was directly attributed to the cytotoxicity of these BAs. Besides, DCA and these relevant hydrophobic BAs can activate nuclear factor kappa-light-chain-enhancer of activated B cells (NF-κB) signaling cascade to induce the production of pro-inflammatory molecules (such as interleukin (IL)-1, IL-8, and tumor necrosis factor-alpha (TNF-α); Miyake et al., 2000; Muhlbauer et al., 2004) and aggravate IL-10 reduction caused by CPT-11 in CD4^+^ T cells (Fang et al., 2016). These reports just support the potential roles of toxic BAs in potentiating the pathological development of mucositis. Moreover, luminal concentrations of BAs are known to exert a cathartic effect by altering salt and water transport (Chadwick et al., 1979; Camilleri et al., 1980; Snape et al., 1980), affecting the expression of various aquaporin water channels (Yde et al., 2016) and stimulating intestinal motility (Alemi et al., 2013). In combination, these effects may contribute to at least certain aspects that patients treated with CPT-11 always suffer from severe late diarrhea.

Co-treatment with HQD could markedly attenuate CPT-11-induced GI toxicity and reverse the alterations of BAs. Despite the fact that BAs pool size and cytoprotective BAs (UDCA, GUDCA, and TUDCA) remained unchanged, it was of interest to note that multiple unconjugated and conjugated hydrophobic BAs were significantly decreased in serum and tissues of rats upon HQD treatment. Besides, the global BAs metabolic profiles in HQD group was more similar to normal control group. Although the mechanisms behind why HQD could regulate the BAs metabolism homeostasis were not solved in this paper, it has been reported that the bioactive constituents of HQD can directly interfere with the normal metabolism of BAs. For example, some natural flavonoids like baicalein and chrysin in *S. baicalensis* were demonstrated to be the pregnane X receptor (PXR) ligands (Dou et al., 2012, 2013). Glycyrrhizic acid, the main active ingredient of *G. uralensis*, can activates farnesoid X receptor (FXR) (Distrutti et al., 2015). Both PXR and FXR can control BAs homeostasis (Modica et al., 2009). Liquiritigenin, an active component of licorice, functions as inducer to increase the protein and mRNA levels of multidrug resistant protein 2 and bile salt export pump and thus influences the transport of BAs (Kim et al., 2009). *P. lactiflora* can also change the serum profiles of BAs by certain unknown mechanisms (Wang et al., 2012). What’s more, most TCM formulas exert multi-targeting actions including the modulation of smooth muscle motility in the GI tract and restoration of intestinal flora (Xiao et al., 2015). Since there are strong and bidirectional interactions between gut microbiome and BAs: microbiota affect the metabolism and function of BAs, while BAs control microbiota overgrowth and protect intestine by their antimicrobial activity (Swann et al., 2011; Sayin et al., 2013; Li and Chiang, 2014). Thus, we speculated that the ability of HQD to restore the homeostasis of BAs may also be linked to its ability to protect the homeostasis of microbiome. These different mechanisms are likely to act synergistically in regulating BAs metabolism Homeostasis.

## Conclusion

In the present study, LC-MS/MS based targeted metabolomics revealed for the first time that the effects of CPT-11-induced intestinal toxicity and HQD’s ability on rat enterohepatic BAs. Compared with total or selected individual BAs level, a whole profile of BAs subspecies may more sensitively reflect the information about intestinal malfunction. Tissues and serum levels of most BAs were significantly decreased after CPT-11 administration, except some hydrophobic BAs. Co-treatment with HQD could markedly attenuate CPT-11-induced GI toxicity and reverse the alterations of BAs. Despite that there was no change in total BAs levels compared with model group, the balance of BAs had shifted leading to decreased toxicity after HQD treatment. Although the mechanisms behind why specific BA was elevate or decreased in diarrhea rats and why HQD could improve the BAs metabolism homeostasis were not solved in this paper, the value of further research was emphasized.

## Author Contributions

XW carried out most of the studies, performed statistical analysis, and wrote the manuscript. DC and XD participated in the animal experiments. JW and WZ provided professional advices. ZZ and FX designed the study and revised the manuscript. All authors have read and approved the final version.

## Conflict of Interest Statement

The authors declare that the research was conducted in the absence of any commercial or financial relationships that could be construed as a potential conflict of interest. The reviewer JW and handling Editor declared their shared affiliation, and the handling Editor states that the process nevertheless met the standards of a fair and objective review.
