# Supplementary material for: HuangQin Decoction Attenuates CPT-11-Induced Gastrointestinal Toxicity by Regulating Bile Acids Metabolism Homeostasis
Source: Front Pharmacol. 2017 Mar 30;8:156. doi: 10.3389/fphar.2017.00156 (PMC5371663; doi:10.3389/fphar.2017.00156)
Supplement: Supplementary file 1 [file Presentation_1.pdf]

Supplementary Material

HuangQin Decoction Attenuates CPT-11-Induced Gastrointestinal Toxicity by Regulating Bile Acids Metabolism Homeostasis

Xu Wang[1,2], Dong-ni Cui[1,2], Xiao-min Dai[1,2], Jing Wang[1,2], Wei Zhang[3], Zun-jian Zhang[1,2], Feng-guo Xu[1,2*]

[1]Key Laboratory of Drug Quality Control and Pharmacovigilance, Ministry of Education (MOE), China Pharmaceutical University, Nanjing, China

[2]State Key Laboratory of Natural Medicine, China Pharmaceutical University, Nanjing, China

[3]State Key Laboratory for Quality Research in Chinese Medicines, Macau University of Science and Technology, Taipa, Macau, China

*** Correspondence:** Feng-guo Xu
E-mail: fengguoxu@gmail.com

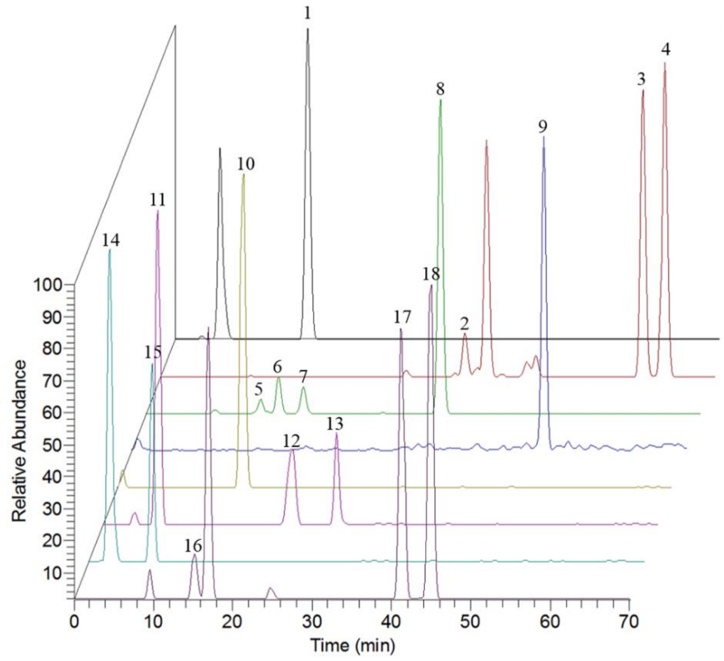

1.GCA 2.UDCA 3.CDCA 4.DCA 5.ω-MCA 6.α-MCA 7.β-MCA 8.CA 9.TLCA
10. IS 11.TUDCA 12.TCDCA 13.TDCA 14.TMCA 15.TCA 16.GUDCA 17.GCDCA 18.GDCA

Supplementary Figure 1. Representative LC-MS/MS chromatograph of 17 BAs determined in serum. The main parameters for MS/MS detection of BAs as well as the internal standard cortisone acetate are summarized in Table.1

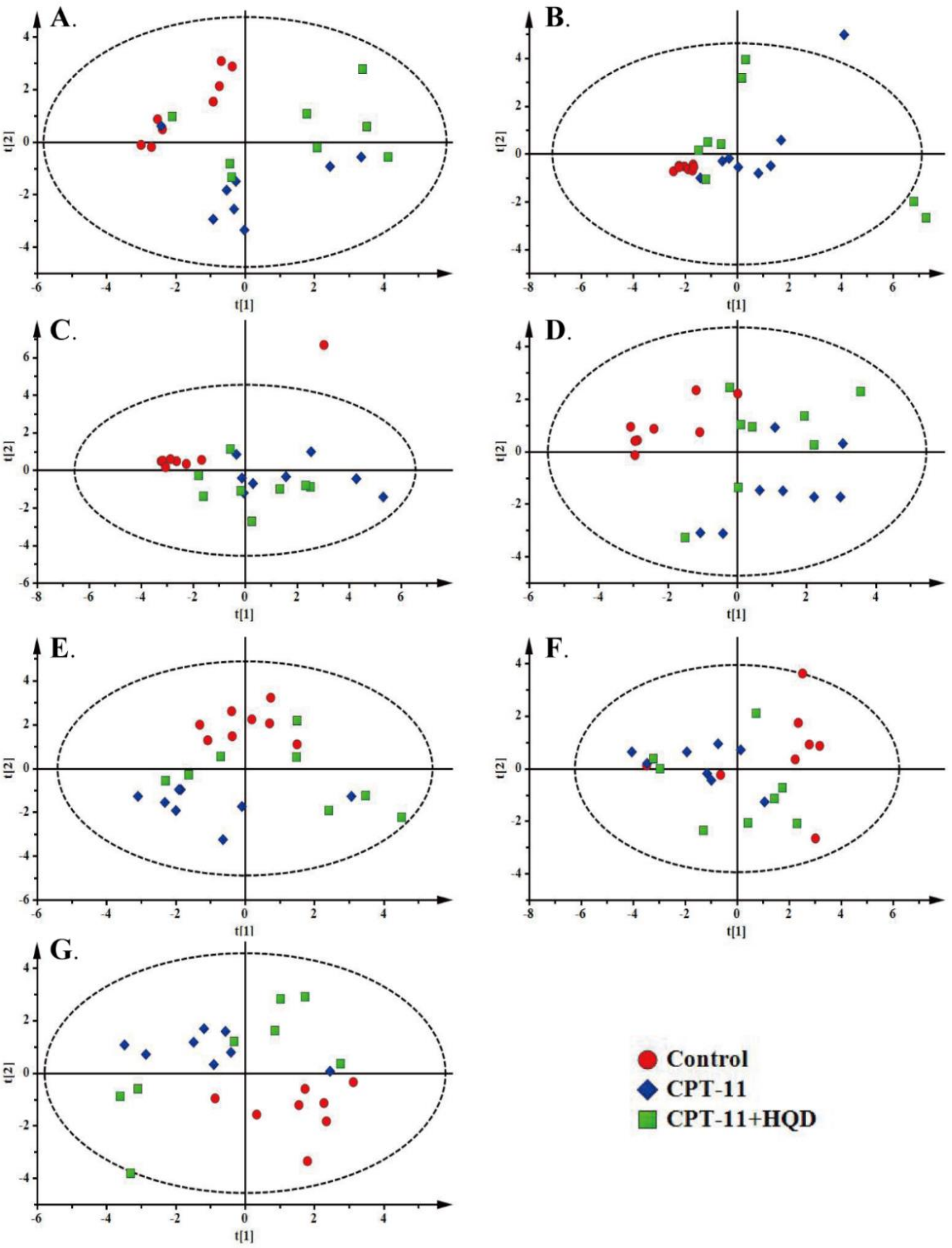

Supplementary Figure 2. PCA models of bile acids profiles.

(A) Serum, R^2X = 0.522, Q^2 = 0.176; (B) Liver, R^2X = 0.625, Q^2 = 0.145; (C) Jejunum, R^2X = 0.591, Q^2 = 0.147; (D) Ileum, R^2X = 0.452, Q^2 = 0.0134; (E) Cecum, R^2X = 0.527, Q^2 = 0.0165; (F) Colon, R^2X = 0.540, Q^2 = 0.192; (G) Rectum, R^2X = 0.502, Q^2 = 0.228.

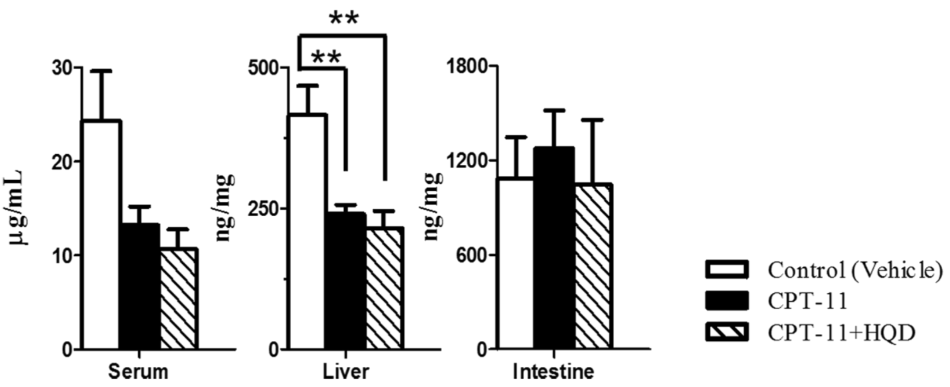

Supplementary Figure 3. Total amount of bile acids in individual compartments.
The bile acids profile in the intestine shows the combined data from five segments.
Mean values ± SD are plotted. ****P<0.01 (Mann-Whitney U test)

Supplementary Table 1. Comparison of BAs levels in biological samples among different groups

Bile acids	Serum		Liver		Jejunum		Ileum		Cecum		Colon		Rectum	
	T *vs* C	H *vs* T	T *vs* C	H *vs* T	T *vs* C	H *vs* T	T *vs* C	H *vs* T	T *vs* C	H *vs* T	T *vs* C	H *vs* T	T *vs* C	H *vs* T
CA	↓***	↑#					↓*	↑#						
GCA														
TCA	↓**		↓**		↓*								↓**	
α-MAC	↓**	↑#												
β-MCA			↑*	↓#		↓#					↑*			
ω-MCA														
TMCA	↓**	↑#					↓*		↓**				↓**	
UDCA	↓*	↑#												
GUDCA														
TUDCA	↓**		↓**				↓*		↓**	↓#			↓*	
CDCA					↑*				↑*		↑*		↑*	↓#
GCDCA			↑*			↓#								
TCDCA						↓#								
DCA	↑**	↓#	↑**	↓#	↑**	↓#	↑*		↑**	↓##	↑***	↓##	↑**	↓##
GDCA	↑***	↓#	↑***	↓#			↑*	↓#	↑**	↓#	↑**	↓##		
TDCA	↑*		↑**	↓##	↑**	↓#	↑*		↑**	↓##	↑***	↓###	↑*	↓##
TLCA	↑*	↓##	↑*	↓#	↑**	↓#	↑**	↓##						

Comparison results are based on the accurate quantitative values of 17 BAs.

C, Control group; T, CPT-11 treated group; H, CPT-11 and HQD co-treated group.

↑ Content increased; ↓ Content decreased; *Significance compared with control group: ***P<0.001, **P<0.01, *P<0.05; #Significance compared with model group: ###P<0.001, ##P<0.01, #P<0.05. (Mann-Whitney U test)
